# GO Trimming: Systematically reducing redundancy in large Gene Ontology datasets

**DOI:** 10.1186/1756-0500-4-267

**Published:** 2011-07-28

**Authors:** Stuart G Jantzen, Ben JG Sutherland, David R Minkley, Ben F Koop

**Affiliations:** 1 Department of Biology & Centre for Biomedical Research, University of Victoria, Victoria, British Columbia, V8W 3N5, Canada

## Abstract

**Background:**

The increased accessibility of gene expression tools has enabled a wide variety of experiments utilizing transcriptomic analyses. As these tools increase in prevalence, the need for improved standardization in processing and presentation of data increases, as does the need to guard against interpretation bias. Gene Ontology (GO) analysis is a powerful method of interpreting and summarizing biological functions. However, while there are many tools available to investigate GO enrichment, there remains a need for methods that directly remove redundant terms from enriched GO lists that often provide little, if any, additional information.

**Findings:**

Here we present a simple yet novel method called GO Trimming that utilizes an algorithm designed to reduce redundancy in lists of enriched GO categories. Depending on the needs of the user, this method can be performed with variable stringency. In the example presented here, an initial list of 90 terms was reduced to 54, eliminating 36 largely redundant terms. We also compare this method to existing methods and find that GO Trimming, while simple, performs well to eliminate redundant terms in a large dataset throughout the depth of the GO hierarchy.

**Conclusions:**

The GO Trimming method provides an alternative to other procedures, some of which involve removing large numbers of terms prior to enrichment analysis. This method should free up the researcher from analyzing overly large, redundant lists, and instead enable the concise presentation of manageable, informative GO lists. The implementation of this tool is freely available at: http://lucy.ceh.uvic.ca/go_trimming/cbr_go_trimming.py

## Background

Transcriptomic experiments conducted using high-density microarrays or RNA-seq often compare two or more states and can generate differentially expressed gene lists comprising hundreds or thousands of genes. These datasets generally require further analysis to identify reliable patterns in expression profiles, such as developmental changes at certain time points, and variable biological processes, such as metabolic pathways. Further analyses such as Gene Ontology (GO) enrichment, pathway enrichment, or clustering methods [1,2] can aid in both the discovery and summarization of important large-scale expression patterns.

GO vocabularies are structured as directed acyclic graphs with a clearly defined hierarchical structure. However, this hierarchy contains an added complexity by allowing terms to have multiple parents, or ascendants [3]. An ascendant and a descendant exist in a defined parent-child relationship and constitute a path through the GO hierarchy, connected by zero or more intermediate GO terms. A gene annotated with any term is also annotated with every term that is an ascendant, or parent term, of the more specific term; each GO category will contain all of the genes from each of its children categories. As a gene will be annotated by a term and every ancestor of this term, terms at a variety of depths in the hierarchy will appear in an enriched GO list, given that the GO tool being used recognizes all levels of annotation for an input gene. In most cases, multiple terms from the same hierarchical path will appear in a significant GO list. These multiple categories of differing specificities are not necessarily problematic. On the contrary, they allow for several levels of interpretation, ranging from specific terms that encompass few genes, to higher-level categories that may describe large-scale effects on the system being studied.

A current area of study to improve GO analysis focuses on the issue of interdependence between terms in the GO hierarchy, the problem being that many tools used to investigate GO enrichment search for enrichment on a term-for-term basis and do not account for correlations among terms along a path in the hierarchy [4]. Due to the detailed structure and incremental specificity of the GO database, as well as the correlation among enriched terms in a path, there will often be instances in which multiple categories from the same path appear in a list and differ only slightly, or not at all in gene content. When this occurs, often the parent term provides no additional information to the researcher or reader, especially when the terms themselves differ only in a small qualifier. An incorrect assumption can be made by the researcher that the multiplicity of similar categories increases the importance of the function or process to which they relate, whereas it is more likely that one group of genes is causing the inclusion of multiple terms. Accordingly, it makes analysis and presentation clearer when closely related terms containing the same genes are removed. However, this term removal can introduce another issue if the subset of terms to be presented is selected mainly due to the specific interest of the researcher. Reducing the size of GO lists in this manner typically uses arbitrary criteria and can be misleading, as the selected subset may not adequately reflect the entire dataset.

Several tools and databases have been developed for the purpose of reducing the inclusion of terms of varying specificity. One type of method proposed to address this issue involves reducing the size of the input database. The Gene Ontology Consortium has produced the GO Slim database, which is a subset comprising more general GO terms [5]. Alternatively, the GO Fat database, developed as part of the Annotation Tool of the DAVID suite of bioinformatics resources, is a subset comprising more specific terms [6]. Both of these methods function by limiting information prior to the enrichment test and therefore do not fully utilize the complete GO database.

An alternative method to reduce the resulting amount of enriched terms is through the use of multiple test corrections (MTC) during GO enrichment tests. These methods not only reduce the output dataset, but can also reduce false positives. Goeman and Mansmann [7] present a method that uses the structure of the GO hierarchy to perform MTC in a top-down, bottom-up, or bi-directional 'focus level' manner (working in both directions from a user-defined level of the hierarchy), depending on the desired objective of the researcher.

Other methods use the full GO database for enrichment analysis and remove terms or modify relationships between terms during the enrichment test to address the interdependency issue [4,8]. Alexa et al. [8] present iterative bottom-up algorithms that either remove genes from parent categories when a child in the same path is significantly enriched (elim), or reduce the weight of genes in categories that have more significant neighbours in its path (weight). Grossman et al. [4] present the parent-child intersection/union algorithms to reduce the inheritance problem by investigating enrichment in the context of parent-child relationships; a term is significant only if the enrichment is due to its own enriched gene set, rather than due to genes inherited from other categories.

Alterovitz et al. [9] have provided a method to investigate categories across a specified information level of the GO hierarchy by generating a numerical value of the "information content" of each GO term and thus could be used to reduce the size of the output dataset.

Clustering GO terms with similar content has also been implemented in both the GOstat and DAVID tools [10,11]. Clustering GO terms using these tools increases an understanding of commonalities between terms due to containment of similar sets of genes. However, these methods do not select or remove terms from the total list of enriched terms, and therefore the large dataset remains. Additionally, these clusters do not use the GO hierarchy or follow the parent-child path, but rather cluster based on gene content alone.

We have developed a simple, systematic method called GO Trimming for removal of redundancy from a GO category list after enrichment scores are given to terms, and is independent of any statistical package or analysis method. This method consists of an algorithm that is executed in two phases. We present an example of this process performed on a sample GO dataset, and highlight the categories that would be removed by the GO Trimming process according to different levels of stringency. Additionally, we compare GO Trimming to several of the aforementioned approaches performed on a second published dataset.

### Algorithm

**Figure 1:**
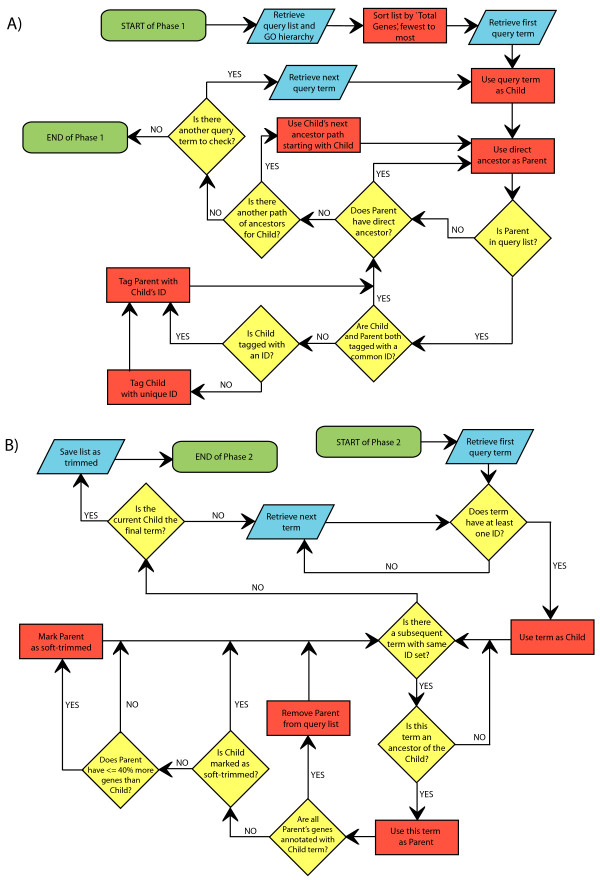
**GO Trimming algorithm flowchart**. A) Phase 1 of GO Trimming: identification of parent-child relationships in GO hierarchy. B) Phase 2 of GO Trimming: strict and soft trimming using 0% and 40% uniqueness thresholds. Green boxes represent start and endpoints for the algorithm. Blue parallelograms represent input and output steps. Red rectangles represent an action required by the user and yellow diamonds represent questions that determine the flow of the algorithm. Input for the algorithm is the query list (list of enriched GO terms) and the GO tree (hierarchy of all GO terms). Output is a list of GO terms with soft and strict trimmed terms removed.

The GO Trimming algorithm is fully described by the flowchart in Figure 1 and is outlined here. GO Trimming consists of two phases, each requiring one pass through the list of significant GO terms. In the first phase, terms are connected to all other terms that share a common path by labelling with common identifiers (Figure 1A). In the second phase, terms are removed from the list based on levels of redundancy between terms found in a given path (Figure 1B).

The list of significantly enriched GO terms is retrieved by the researcher (Figure 1A) from any tool that performs statistical testing on GO categories (e.g. GeneSpring GX (Agilent, Santa Clara, CA); GOstat [10]). In addition, each term must have the following associated information: the number of differentially regulated genes annotated with the term (labelled "Diff. Genes" in Table 1) and the total number of genes annotated with the GO term (e.g. all genes on a microarray annotated with the term; labelled "Total Genes" in Table 1). Since these are the numbers used to test for enrichment, these values should be available from the same tool used to generate the list of significant terms. The hierarchical relationships of these categories are then obtained from the GO tree whether from within a software package like GeneSpring GX, or from the web platform AmiGO [12] or a downloadable database [13]. GO categories are considered query terms and are sorted in ascending order of "Total Genes". This order of query terms is maintained throughout both phases. Care should be taken after sorting to ensure that in cases of equal totals for a pair of terms in a parent-child relationship, parents are ranked after children in the list to avoid missed identification.

**Table 1:** Sample Gene Ontology terms and GO Trimming results

ID	GO Accession	GO Term	p-value	Diff. Genes	Total Genes
1	GO:0031532	actin cytoskeleton reorganization	0.00338	5	12
2	GO:0009620	response to fungus	3.04E-09	10	12
3	GO:0006037	cell wall chitin metabolic process	3.04E-09	10	12
**3**	**GO:0044036**	**cell wall macromolecule metabolic process**	**3.04E-09**	**10**	**12**
**3**	**GO:0010383**	**cell wall polysaccharide metabolic process**	**3.04E-09**	**10**	**12**
4	GO:0003796	lysozyme activity	3.04E-09	10	12
	GO:0001101	response to acid	3.04E-09	10	12
5	GO:0046578	regulation of Ras protein signal transduction	6.77E-04	6	13
**5**	**GO:0051056**	**regulation of small GTPase mediated signal transduction**	**6.77E-04**	**6**	**13**
6	GO:0008061	chitin binding	1.21E-08	10	13
7	GO:0006032	chitin catabolic process	1.21E-08	10	13
**3**	**GO:0006030**	**chitin metabolic process**	**1.21E-08**	**10**	**13**
8	GO:0004568	chitinase activity	1.21E-08	10	13
9	GO:0003746	translation elongation factor activity	0.00727	5	14
**3**	**GO:0071554**	**cell wall organization or biogenesis**	**3.86E-08**	**10**	**14**
	GO:0008643	carbohydrate transport	0.0226	5	18
10	GO:0005885	Arp2/3 protein complex	0.0226	5	18
***9***	***GO:0006414***	***translational elongation***	***0.00485***	***6***	***18***
11	GO:0045165	cell fate commitment	0.0284	5	19
	GO:0035091	phosphoinositide binding	0.00652	6	19
**7**	**GO:0006026**	**aminoglycan catabolic process**	**2.29E-06**	**10**	**19**
12	GO:0030126	COPI vesicle coat	0.0140	6	22
**12**	**GO:0030663**	**COPI coated vesicle membrane**	**0.0140**	**6**	**22**
***7***	***GO:0000272***	***polysaccharide catabolic process***	***1.36E-06***	***11***	***22***
13	GO:0003007	heart morphogenesis	0.0175	6	23
**12**	**GO:0030660**	**Golgi-associated vesicle membrane**	**0.0215**	**6**	**24**
**12**	**GO:0030137**	**COPI-coated vesicle**	**0.0215**	**6**	**24**
14	GO:0005859	muscle myosin complex	0.0215	6	24
**14**	**GO:0016460**	**myosin II complex**	**0.0215**	**6**	**24**
15	GO:0001725	stress fiber	0.00546	7	24
**15**	**GO:0032432**	**actin filament bundle**	**0.00546**	**7**	**24**
**15**	**GO:0042641**	**actomyosin**	**0.00697**	**7**	**25**
3, 7	GO:0006022	aminoglycan metabolic process	1.04E-05	11	26
16	GO:0030239	myofibril assembly	0.0134	7	28
***16***	***GO:0031032***	***actomyosin structure organization***	***0.00441***	***8***	***29***
17	GO:0016363	nuclear matrix	0.00134	9	30
**17**	**GO:0034399**	**nuclear periphery**	**0.00174**	**9**	**31**
18	GO:0007586	digestion	4.24E-06	13	33
19	GO:0001726	ruffle	0.0146	8	35
20	GO:0005529	sugar binding	6.58E-06	14	39
***3, 7***	***GO:0005976***	***polysaccharide metabolic process***	***2.20E-04***	***12***	***40***
	GO:0005938	cell cortex	0.0407	8	42
***16***	***GO:0010927***	***cellular component assembly involved in morphogenesis***	***0.0174***	***9***	***43***
21	GO:0022627	cytosolic small ribosomal subunit	1.20E-05	15	46
22	GO:0004322	ferroxidase activity	4.18E-07	17	46
**22**	**GO:0016724**	**oxidoreductase activity, oxidizing metal ions, oxygen as acceptor**	**4.18E-07**	**17**	**46**
***6***	***GO:0030247***	***polysaccharide binding***	***7.26E-05***	***14***	***47***
**6**	**GO:0001871**	**pattern binding**	**7.26E-05**	**14**	**47**
**22**	**GO:0016722**	**oxidoreductase activity, oxidizing metal ions**	**1.18E-06**	**17**	**49**
23	GO:0022625	cytosolic large ribosomal subunit	0.0428	9	50
***7***	***GO:0016052***	***carbohydrate catabolic process***	***6.10E-05***	***15***	***52***
4, 8	GO:0004553	hydrolase activity, hydrolyzing O-glycosyl compounds	5.05E-05	16	57
***21***	***GO:0015935***	***small ribosomal subunit***	***1.58E-05***	***17***	***58***
24	GO:0005200	structural constituent of cytoskeleton	0.0210	11	59
***23***	***GO:0015934***	***large ribosomal subunit***	***0.0328***	***11***	***63***
***19***	***GO:0031252***	***cell leading edge***	***0.0364***	***11***	***64***
**4, 8**	**GO:0016798**	**hydrolase activity, acting on glycosyl bonds**	**2.74E-04**	**16**	**65**
6, 20	GO:0030246	carbohydrate binding	1.64E-04	18	75
***2***	***GO:0051707***	***response to other organism***	***0.00451***	***15***	***76***
21, 23	GO:0022626	cytosolic ribosome	9.78E-06	24	98
25	GO:0016791	phosphatase activity	0.0314	17	111
16	GO:0032989	cellular component morphogenesis	0.0160	18	111
**25**	**GO:0016311**	**dephosphorylation**	**0.0365**	**17**	**113**
26	GO:0003735	structural constituent of ribosome	3.52E-06	28	118
21, 23	GO:0033279	ribosomal subunit	5.92E-06	28	121
1, 16	GO:0030036	actin cytoskeleton organization	0.00597	21	124
21, 23	GO:0044445	cytosolic part	3.27E-05	27	125
7	GO:0009057	macromolecule catabolic process	0.0131	21	133
***25***	***GO:0042578***	***phosphoric ester hydrolase activity***	***0.0457***	***20***	***142***
	GO:0006955	immune response	0.0486	20	143
***1, 16***	***GO:0030029***	***actin filament-based process***	***0.0189***	***22***	***146***
27	GO:0005615	extracellular space	0.00507	24	146
***21, 23***	***GO:0005840***	***ribosome***	***8.61E-05***	***29***	***146***
19	GO:0042995	cell projection	0.00775	24	151
10, 14, 15	GO:0015629	actin cytoskeleton	0.0282	24	169
***1, 16***	***GO:0007010***	***cytoskeleton organization***	***0.0115***	***29***	***198***
3, 7	GO:0005975	carbohydrate metabolic process	0.0121	31	216
9	GO:0006412	translation	4.07E-04	38	229
***27***	***GO:0044421***	***extracellular region part***	***0.0135***	***33***	***235***
21, 23	GO:0030529	ribonucleoprotein complex	0.00101	43	281
24, 26	GO:0005198	structural molecule activity	2.31E-05	53	316
21, 23	GO:0005829	cytosol	0.00275	47	330
27	GO:0005576	extracellular region	0.00841	46	341
9	GO:0003723	RNA binding	5.39E-04	53	356
***9***	***GO:0003676***	***nucleic acid binding***	***0.00866***	***73***	***588***
13, 28	GO:0007275	multicellular organismal development	0.0203	89	764
10, 14, 15, 21, 23	GO:0043232	intracellular non-membrane-bounded organelle	0.0186	95	819
**10, 14, 15, 21, 23**	**GO:0043228**	**non-membrane-bounded organelle**	**0.0186**	**95**	**819**
11, 13, 16, 28	GO:0032502	developmental process	0.0307	105	934
18, 28	GO:0032501	multicellular organismal process	0.0273	119	1067

List of experimentally-derived Gene Ontology terms significantly enriched (p-value ≤ 0.05; no MTC) by the differential gene list. Columns show identifiers for hierarchical connections, GO accession numbers, GO term descriptors, enrichment p-values, numbers of genes in differentially regulated gene list annotated with GO term, and total numbers of genes on microarray annotated with GO term. Terms in plain text are retained after GO Trimming with all uniqueness threshold values. Bolded terms are removed with a 0% threshold. Bolded and italicized terms are additionally removed when 40% is used as uniqueness threshold for GO Trimming, and bolded, italicized and underlined terms are additionally removed with a 50% threshold.

For any set of genes annotated with a common GO term, it can be said that this GO category contains this set of genes. For each term in the list of GO categories, all ancestors of the term (i.e. having parent-child relationships with the term) are examined, again starting with the term containing the fewest genes and moving towards broader terms. All parent-child paths are labelled with unique identifiers to mark the hierarchical connections between terms. Two terms may have a common parent, so each of these two children will be labelled with different identifiers, and the parent will be labelled with both identifiers. A term may have no parents present in the GO list, and therefore will have no identifier.

Once the entire list has been processed in the first phase, two types of trimming can be applied (Figure 1B). The first more strict approach removes terms that are entirely redundant. The second approach, soft trimming, uses more relaxed stringency, and terms that are largely redundant can be removed. A uniqueness threshold was designed to filter terms based on the respective gene sets contained by the parent and child categories. A value of 0% is used for the strict approach and a value of 40% is used for the soft trimming approach. If the parent category contains the same set of genes as the child category (i.e. parent contains 0% unique genes), it is deemed fully redundant and removed from the strict list (and soft list). With respect to the soft trimming threshold, if the parent term contains 40% or fewer additional genes than the child term (e.g. the child term contains ten genes; the parent term contains these ten plus an additional four), the parent term is removed from the soft trimmed list. In both of these examples, the more specific child category is retained.

If a category is involved in multiple paths in the list and so has more than one identifier, it can only be removed if a descendant shares all identifiers (i.e. has the same ID set) and indicates that the parent category is redundant. When both soft and strict trimming are performed concurrently, soft trimmed terms should not be removed from the list until the end of the process as they may still be used in the strict trimming approach. Note that some IDs may be processed multiple times through the course of the list with different untrimmed terms as the child. This allows for broader terms to be trimmed based on the representation of intermediate terms. During the second phase, it is still important to check for ancestry before trimming, since the structure of the GO hierarchy (i.e. a term can have multiple parents) may allow two terms to have the same ID set, yet not be in a parent-child relationship. As a final note, the 40% value is somewhat arbitrary, and can be raised or lowered, but provides a cut-off that can be used to eliminate terms that seem to be generally unworthy of separate discussion from related terms. Once trimming is complete, all terms that were soft or strict trimmed can be fully removed from the GO list and the reduced list can be presented as fully processed.

### Testing

We present here an example of the use of this method in removing redundant terms from an enriched Gene Ontology term list. This sample dataset was taken from a recent experiment exploring the transcriptional effects of sea lice (*Lepeophtheirus salmonis*) on pink salmon (*Oncorhynchus gorbuscha*) [14]. Although the parameters for the data presented here are slightly different from those for the GO lists shown in this earlier experiment, the biological question and the majority of the information remains the same. An initial list of 90 GO terms (Table 1) were enriched from an input list of 3388 differentially regulated entities. GeneSpring GX 11.0 was used to determine GO enrichment and the hierarchical relationships between enriched terms; the GO database used was from November 04, 2010.

Transcriptomic analysis can typically result in many enriched Gene Ontology terms, and in an attempt to reduce the number of terms enriched by chance, and to present a more manageable example dataset, only GO terms containing 5 or more differentially expressed genes were retained. This pre-filtering was done simply by imposing a threshold on the "Diff. Genes" value in a spreadsheet. This is an independent step from GO Trimming and should not have a substantial influence on the procedure. The DAVID tool [11] for finding enrichment offers a gene count threshold as well (default is 2) for the reason that terms with very few genes are less trustworthy as real trends.

Through the GO Trimming process with 0%, 40%, and 50% uniqueness thresholds, the list was substantially reduced (Table 1), with 19 of 90 terms identified as completely redundant (0% threshold; bolded text). With the use of a 40% soft trimming threshold, another 15 terms were found to be largely redundant (bolded and italicized text). To show the relative flexibility of the threshold value, we also performed the procedure with a threshold of 50% (bolded, italicized and underlined text). With this reduced stringency, only two additional terms were removed from the list when compared with the list after the use of the 40% threshold. Note that several of the most specific and the most general terms are retained, and many of intermediate specificity are discarded.

Looking at the list trimmed using the conservative approach (0% threshold), 19 of 90 terms were shown to be redundant. For example, "oxidoreductase activity, oxidizing metal ions" and "oxidoreductase activity, oxidizing metal ions, oxygen as acceptor" are removed and the term "ferroxidase activity" is retained.

With slightly less stringency, we can remove a number of terms that offer little additional information to the analysis. For example, "polysaccharide catabolic process" adds only one gene to the set of those annotated with the term "chitin catabolic process".

Highly similar terms that remain in the list are often a result of being from different GO domains, such as Biological Process and Molecular Function, the top-level categories of "chitin catabolic process" and "chitinase activity", respectively. Also "sister" terms that appear quite similar but are not in a parent-child relationship (e.g. "cell wall chitin metabolic process" and "chitin catabolic process") cannot be eliminated because they are from different hierarchical paths, and therefore may refer to distinct processes or functions.

In addition to this sample dataset, we performed a comparison of GO Trimming with other methods that attempt to ease interpretation or take into account the interdependencies in the hierarchy during enrichment testing. For this comparison, zebrafish (*Danio rerio*) was deemed to be a suitable organism of study because each of the methods we wished to compare provided the ability to use a ZFIN identifier [15] in annotating genes with GO terms. Accordingly, we found a microarray experiment studying hypoxia in *D. rerio *[16] that resulted in a large number of differentially expressed genes using a well annotated array [17]. Of 1520 significantly differentially regulated entities, 1017 had official gene symbols which were used to link to ZFIN IDs in the GO Consortium's ZFIN annotation file (May 27, 2011) [18]. 617 genes had a corresponding ZFIN ID with GO annotation. This set of 617 ZFIN IDs was the sample dataset or the list of differentially regulated genes. In the same manner, of 42990 entities on the whole array, 24888 had a gene symbol, of which 12674 had a linked ZFIN ID associated with GO annotation. This was the population or total set of genes.

It is apparent that level of annotation and choice of statistical test have a large influence on the results of enrichment testing. For example, GOstat uses a χ^2 ^test and does not permit custom annotation files [10]. Therefore, the default ZFIN annotation database was used, which appeared to have annotation for all but 17 terms in the sample list. This resulted in a large difference in significant terms when compared to the traditional term-for-term method employed in the Ontologizer (175 vs. 236 terms; p-value ≤ 0.1) [19]. Additionally, GOstat did not include an option for a 0.05 p-value cut-off. The DAVID tool also used its own associations with ZFIN IDs, resulting in a different list of enriched terms.

Due to the differences in statistical tests and annotation, we restricted the formal comparison to those tests which could be performed using the Ontologizer [19], including elim, weight, and the parent-child methods [4,8]. The traditional term-for-term method was also included, and the output of this method was used as the input list for the GO Trimming process. A p-value cut-off of 0.05 was employed, and no MTC was used, as the impact of MTC may differ between methods. In this sample dataset, no gene count threshold was used.

The significantly enriched terms (p-value ≤ 0.05) resulting from each method are presented in Additional file 1. Any term enriched through one or more methods is listed in the table, and the enrichment is represented by a p-value. In summary, the term-for-term method resulted in the largest number of enriched terms (147), followed by elim (137), weight (86), GO Trimming (80), parent-child union (78), and parent-child intersection (46). The term-for-term output included all but 24 terms; the elim, parent-child intersection and parent-child union methods resulted in the inclusion of some additional terms.

We used the *D. rerio *dataset on two other methods: GOstat clustering [10] and DAVID clustering [11] (data not shown). Instead of reducing the number of terms produced by enrichment testing, the significant terms are clustered into groups that aim to improve interpretation of the results.

## Discussion

GO Trimming was designed with the idea of reducing redundancy while fully utilizing the size and detail of the GO database. We believe this method is versatile and can be tailored to the needs of researchers while still being systematic by nature so that it can be easily integrated into an analysis workflow.

The first sample dataset (Table 1) provides a good example of what GO Trimming does and does not do. In this example, there is no real biological information lost to the researcher through the trimming process. Nor in fact, is there any biological information added that was not already present. The p-value of individual terms is not adjusted. Neither does GO Trimming serve to intentionally eliminate false positives. In fact this process is independent of MTC, as MTC can be applied during the enrichment testing, and GO Trimming may be performed on the output list.

After trimming, the information in Table 1 becomes more focused and balanced, making interpretation easier. Redundant terms no longer overwhelm the list as in the cases of IDs 3 and 12. Terms in a unique path, such as "immune response", a biologically important term, do not become lost in long lists [20]. Once redundant terms are removed, such as the terms related to polysaccharide, actin, and oxidoreductase functions, it becomes easier to consider and present the entire list, and terms such as "ruffle", which was important in the context of the experiment [14], can come to the forefront. Additionally, it becomes more feasible to present the list in the manuscript, instead of picking out only a select few to discuss. Ultimately it is up to the researcher how to interpret and discuss results, however GO Trimming provides a way to assist in this process.

Although the GO Trimming output list is easier to manage and interpret than an untrimmed list, it is important to use a non-destructive workflow where the full list of terms is retained for potential further exploration of specific results. We encourage researchers to append the full GO category list to published articles as supplemental documents, but for general table and text presentation, the trimmed list should be used.

Furthermore, working with GO lists in this way can familiarize the researcher to the general patterns and functions present in the data. Adding identifiers to parent-child relationships in the GO lists not only assists with the trimming process, but also connects terms with related functions and properties, allowing for ease in locating reoccurring themes. For example in the sample dataset above (Table 1), "actin cytoskeleton reorganization" and "myofibril assembly" share the parent "actin cytoskeleton organization and biogenesis".

Terms with multiple identifiers represent a synthesis of information, as they represent the union of multiple paths in the GO hierarchy. Alternatively, terms without identifiers are those that have no parents or children present in the list. This in itself provides some information about the term and the associated genes. Increased understanding of the connections between terms will allow for increased comprehension of the processes under investigation.

Understanding these connections between terms is a similar benefit to that offered by GOstat or DAVID clustering [10,11]. These clustering methods cannot be directly compared to GO Trimming, primarily due to the structure of the output. Instead of reducing terms in the output lists, terms are organized into categories that share information. One benefit of GO Trimming is the more manageable presentation. With clustering, either the researcher can present all terms, which can result in very sizeable lists, or the researcher can select a representative from each cluster to present. If a representative is selected, such as the most significant term, a main function or process may be preserved, but other valuable information could be lost.

With respect to GOstat specifically, the clustering method is highly inclusive while creating clusters. Any term containing a subset of genes annotated to another term will be clustered together. This does not take into account the GO hierarchy, which in some cases may be beneficial, in that closely related terms under difference roots (e.g. "biological process" and "molecular function") can be clustered together. However, this can also result in more disparate terms being grouped into a cluster, simply by containing common genes. Regarding the clustered output of DAVID, non-significant terms appear to be included, which could be removed after clustering. Also, clusters can consist entirely of terms that are essentially redundant. Overall, these clustering methods can aid in interpretation of results, however the problem of redundancy is not addressed, the GO hierarchy is not taken into account, and clusters can be too inclusive.

The comparison between GO Trimming and methods employed by the Ontologizer [19] provides insight into benefits and drawbacks of each method. There are a few trends identifiable based on specificity of enriched terms (Additional file 1). It is apparent that elim and weight methods produce more specific enriched terms and fewer general terms. This is not necessarily negative, since specific terms are arguably more interesting and informative to a researcher, although it can be informative to examine higher level terms. One major benefit of Gene Ontology is the ability to identify functions and processes at different depths [21]. Furthermore, there appear to be many redundant terms enriched using the elim and weight methods through the Ontologizer (e.g. many parents of "negative regulation of neutrophil chemotaxis").

The parent-child union method seems to result in a much lower level of redundancy and provides a lighter but still informative set of terms. The method behind it appears to be strong, in checking for enrichment of a term in the context of its parent(s), but it too results in some redundancy in the list (e.g. "branching morphogenesis of a tube", "morphogenesis of branching epithelium", "morphogenesis of a branching structure"; Additional file 1). The parent-child intersection method seems to result in much fewer terms being enriched, and may be too stringent, resulting in information being lost. These methods are more directed towards decorrelating terms from each other so as to minimize the effect of genes being inherited through the hierarchy and to reduce false positives [4].

Compared to these other methods, GO Trimming is highly effective at reducing redundancy at both specific and general levels. For example, regarding the parents of "negative regulation of neutrophil chemotaxis", GO Trimming removes 10-12 closely related terms, many of which are included in the results of the other methods (with the exception of parent-child intersection). At the general level, many parent terms of "ATP binding" are removed by GO Trimming. While it does not address the issue of false positives, GO Trimming specifically targets and reduces redundancy without losing information, which may occur through more stringent methods.

## Conclusion

We have a developed a novel and important method for systematically reducing redundancy in Gene Ontology datasets. The simplicity of this method allows for ease of incorporation into a typical transcriptomic workflow, while still using the full structure of the GO hierarchy. It focuses on improving interpretation and presentation, and compares well against other GO enrichment methods that take into consideration interdependencies in the GO hierarchy. With the exception of the stringent parent-child intersection method, the resulting list of terms contains the least redundancy, offering a cleaner, more focused representation of the dataset. With this method, researchers are able to analyze and present terms in a way that will provide the most information about the genes and systems being studied.

## Competing interests

The authors declare that they have no competing interests.

## Authors' contributions

SGJ and BJGS developed the algorithm and drafted the manuscript. DRM implemented the algorithm. BFK provided discussion of ideas and assisted in preparing the manuscript. All authors read and approved the final manuscript.

## Supplementary Material

Additional file 1 **Supplementary Table 1. Comparison of GO Trimming and enrichment methods on *D. rerio *dataset**. Using the Ontologizer tool, a number of methods produce statistically enriched GO terms (p-value ≤ 0.05; no MTC) from a set of 617 differentially regulated genes. The union of GO terms enriched by one or more methods is presented, along with the p-values each method produced for enriched terms, sorted by "Total Genes" from specific terms to general terms. GO Trimming was performed on the output of the traditional term-for-term method using a 40% soft trimming threshold. P-values from the term-for-term method are presented for those terms retained by the GO Trimming method. Click here for file

## References

[B1] Ashburner M Ball C Blake J Botstein D Butler H Cherry J Davis A Dolinski K Dwight S Eppig J Harris M Hill D Issel-Tarver L Kasarskis A Lewis S Matese J Richardson J Ringwald M Rubin G Sherlock G Consortium GO Gene Ontology: tool for the unification of biology Nat Genet 2000 25 1 25 29 10.1038/75556 10802651 PMC3037419

[B2] Kanehisa M Goto S KEGG: Kyoto Encyclopedia of Genes and Genomes Nucleic Acids Res 2000 28 1 27 30 10.1093/nar/28.1.27 10592173 PMC102409

[B3] Ashburner M Ball C Blake J Butler H Cherry J Corradi J Dolinski K Eppig J Harris M Hill D Lewis S Marshall B Mungall C Reiser L Rhee S Richardson J Richter J Ringwald M Rubin G Sherlock G Yoon J Consortium GO Creating the gene ontology resource: Design and implementation Genome Res 2001 11 8 1425 1433 10.1101/gr.180801 11483584 PMC311077

[B4] Grossmann S Bauer S Robinson PN Vingron M Improved detection of overrepresentation of Gene-Ontology annotations with parent-child analysis Bioinformatics 2007 23 22 3024 3031 10.1093/bioinformatics/btm440 17848398

[B5] GO Slim http://www.geneontology.org/GO.slims.shtml

[B6] Dennis G Sherman B Hosack D Yang J Gao W Lane H Lempicki R DAVID: Database for annotation, visualization, and integrated discovery Genome Biol 2003 4 9 R60 10.1186/gb-2003-4-9-r60 PMC193660 12734009

[B7] Goeman JJ Mansmann U Multiple testing on the directed acyclic graph of gene ontology Bioinformatics 2008 24 4 537 544 10.1093/bioinformatics/btm628 18203773

[B8] Alexa A Rahnenfuehrer J Lengauer T Improved scoring of functional groups from gene expression data by decorrelating GO graph structure Bioinformatics 2006 22 13 1600 1607 10.1093/bioinformatics/btl140 16606683

[B9] Alterovitz G Xiang M Mohan M Ramoni MF GO PaD: The gene ontology partition database Nucleic Acids Res 2007 35 Sp. Iss. SI D322 D327 17098937 10.1093/nar/gkl799 PMC1669720

[B10] Beissbarth T Speed TP GOstat: find statistically overrepresented Gene Ontologies within a group of genes Bioinformatics 2004 20 9 1464 1465 10.1093/bioinformatics/bth088 14962934

[B11] Huang DW Sherman BT Tan Q Kir J Liu D Bryant D Guo Y Stephens R Baseler MW Lane HC Lempicki RA DAVID Bioinformatics Resources: expanded annotation database and novel algorithms to better extract biology from large gene lists Nucleic Acids Res 2007 35 W169 W175 10.1093/nar/gkm415 17576678 PMC1933169

[B12] AmiGO http://amigo.geneontology.org/cgi-bin/amigo/go.cgi

[B13] GO Database Downloads http://www.geneontology.org/GO.downloads.database.shtml

[B14] Sutherland BJG Jantzen SG Sanderson DS Koop BF Jones SRM Differentiating size-dependent responses of juvenile pink salmon (*Oncorhynchus gorbuscha*) to sea lice (*Lepeophtheirus salmonis*) infections Comp Biochem Physiol Part D Genomics Proteomics 2011 6 2 213 223 10.1016/j.cbd.2011.04.001 21543273

[B15] Bradford Y Conlin T Dunn N Fashena D Frazer K Howe DG Knight J Mani P Martin R Moxon SA Paddock H Pich C Ramachandran S Ruef BJ Ruzicka L Bauer Schaper H Schaper K Shao X Singer A Sprague J Sprunger B Van Slyke C Westerfield M ZFIN: enhancements and updates to the zebrafish model organism database Nucleic Acids Res 2011 39 suppl 1 D822 D829 21036866 10.1093/nar/gkq1077 PMC3013679

[B16] Martinovic D Villeneuve DL Kahl MD Blake LS Brodin JD Ankley GT Hypoxia alters gene expression in the gonads of zebrafish (*Danio rerio*) Aquat Toxicol 2009 95 258 272 10.1016/j.aquatox.2008.08.021 18977541

[B17] Platform GPL6563: Agilent-015064 http://www.ncbi.nlm.nih.gov/geo/query/acc.cgi?acc=GPL6563

[B18] GO Current Annotations http://www.geneontology.org/GO.downloads.annotations.shtml

[B19] Bauer S Grossmann S Vingron M Robinson PN Ontologizer 2.0 - a multifunctional tool for GO term enrichment analysis and data exploration Bioinformatics 2008 24 1650 1651 10.1093/bioinformatics/btn250 18511468

[B20] Toronen P Pehkonen P Holm L Generation of Gene Ontology benchmark datasets with various types of positive signal BMC Bioinformatics 2009 10 319 10.1186/1471-2105-10-319 19811632 PMC2762998

[B21] Khatri P Draghici S Ontological analysis of gene expression data: current tools, limitations, and open problems Bioinformatics 2005 21 18 3587 3595 10.1093/bioinformatics/bti565 15994189 PMC2435250

